# Religiosity and Health-Related Risk Behaviours in a Secular Culture—Is there a Correlation?

**DOI:** 10.1007/s10943-019-00919-2

**Published:** 2019-09-27

**Authors:** Nanna Herning Svensson, Niels Christian Hvidt, Susanne Pagh Nissen, Maria Munch Storsveen, Elisabeth Assing Hvidt, Jens Søndergaard, Trine Thilsing

**Affiliations:** Research Unit for General Practice, Department of Public Health, University of Southern Denmark, J. B. Winsløws Vej 9A, 5000 Odense, Denmark

**Keywords:** Religiosity, Secular, Lifestyle, Risk behaviour

## Abstract

In the present study, we examine the correlation between religiosity and health-related risk behaviours among citizens aged 29–60 based on a cross-sectional survey in Denmark, known for its more secular culture. Health-related risk behaviours such as smoking and alcohol intake are known to increase the risk of developing one or more chronic or life-threatening diseases. In this study religiosity, in a random sample of Danes, seems to be associated with healthier lifestyle, such as a healthier dietary pattern and less smoking, as is found in more religious cultures. Our study suggests that religious practice among Danish citizens seems to be correlated with health behaviours and that healthcare professionals should pay more attention to the connection between religiosity and health.

## Introduction

Individuals with health-related risk behaviours have an increased risk of developing one or more chronic or life-threatening diseases (National Center for Chronic Disease Prevention and Health Promotion 2014). The most important risk factors comprise unhealthy diet, smoking, increased alcohol intake, physical inactivity and overweight (Habib and Saha 2010). For example, in Denmark alone it is estimated that every third death is caused by smoking (Sundhedsstyrelsen 2018), and smokers are much more likely to develop cancer, cardiovascular diseases and chronic obstructive pulmonary disease (COPD) (National Center for Chronic Disease Prevention and Health Promotion 2014). By eliminating modifiable risk factors such as unhealthy lifestyle, it is possible to prevent 80% of heart and/or circulatory diseases (Nielsen et al. 2017). For example, improving dietary habits by an intake of fruit and vegetables of 500 grams per day is estimated to reduce the cardiovascular death rate by approximately 17% (Juel et al. 2006).

The burden of the above-mentioned diseases may also be influenced by religious belief and practice as research has shown a positive correlation between religiosity and overall health status (Koenig et al. 2012; Lau Caspar Thygesen et al. 2012a). In addition, religiosity has been associated with a lower incidence of cancer (Thygesen et al. 2012a), cardiovascular diseases (Sorensen et al. .^2011^), mental disorders (Thygesen et al. 2013) and suicide (Koenig et al. 2012). Evidence based on systematic reviews comprising several longitudinal studies suggests that the positive impact of religiosity on health is mediated through better lifestyle (Koenig et al. 2012). People who participate in religious activities (e.g. prayer or church attendance) are less likely to engage in risk behaviour such as smoking and excessive alcohol consumption compared to non-religious individuals (Bailey et al. 2015; Brown et al. 2014; Kobayashi et al. 2015; Koenig et al. 2012; Shmueli and Tamir 2007). Furthermore, religious individuals are more likely to have a healthy dietary pattern (Thygesen et al. 2012b) and to be more physically active compared to non-religious individuals (Kim and Sobal 2004; Strawbridge et al. 2001). Research on the associations between religiosity and body weight shows inconsistent results, also within the same religious affiliation, but most of the studies find that religiosity is associated with higher body weight (Yeary et al. 2017).

Studies investigating the correlation between religiosity and health-related risk behaviours in a young to middle-aged secular population are few. Denmark may be considered a country with some levels of secularity, meaning that institutionalized religious belief and practice are retreating in public space (Assing Hvidt et al. 2012; Zuckerman 2008). Denmark features one of the world’s largest paying membership of any church (The Lutheran Church of Denmark) and over 70% call themselves “believers” (Andersen and Lüchau 2011), but only few practice this faith in the form of frequent church attendance. Spirituality and faith do not seem to be absent, but rather detraditionalized, individualized and privatized. Most of the research concentrating on the associations between religion and health has been conducted in the USA or among religious minority groups such as the Seventh Day Adventists and Baptists (Holt et al. 2017; Kim and Sobal 2004; Shmueli and Tamir 2007; Lau Caspar Thygesen et al. 2012b), where the degree and character of religious belief and practice is nowhere comparable to the religious characteristics of the general Danish population (la Cour 2004). Previous research in Denmark concerning religion and health has primarily been focusing on twins, the older segment of the population (+50 years) or individuals suffering from serious illness (Ahrenfeldt et al. 2016, 2017; la Cour 2008). A younger segment of the Danish population has not been adequately studied; therefore, the present study seeks to fill in this knowledge gap.

The aim of this study is thus to investigate the correlation between religiosity (measured by church/mosque attendance and prayer/meditation practice) and health-related risk behaviours such as unhealthy diet, daily smoking, alcohol consumption above the high-risk limit, physical inactivity and overweight (BMI ≥ 25) in a cross-sectional study among 29–60-years-old Danes.

## Methods

### Setting and Study Population

Data are retrieved from the pilot project *Early Detection and Prevention* (TOF), conducted in the Region of Southern Denmark. The aim of the TOF project is to systematically identify individuals at high-risk of lifestyle-related disease and individuals with risk behaviours, and to provide targeted and coherent preventive services. Full information on the project is available in the following article (Larsen et al. 2018a, b). The project was conducted in two Danish municipalities in the Region of Southern Denmark (Varde and Haderslev), with participation of 47 general practitioners. A random sample of 8814 patients from the GPs’ patient population aged 29–60-years-old were invited to participate, and patients consenting to participate were asked to fill in a questionnaire with questions on lifestyle as well as on religiosity.

### Religiosity

Religiosity was assessed by two different questions, namely religious attendance in churches or mosques and prayer/meditation practice. Attendance was measured by the question, “*How often do you go to church, mosque or other religious community? Please do not include weddings, funerals and christening”* with the following response categories 1: “*At least once a month*”, 2: “*Less than once a month*”, 3: “*On special occasions (like Christmas and Easter)*”, 4: “*Never, almost never*” and 5: “*Do not know”.* Prayer/meditation practice was measured by the question, “*It happens that I pray, meditate or the like”* with the following response categories 1: “*To a very great extent*”, 2: “*To a great extent*”, 3: “*To some extent*”, 4: “*Not at all*” and 5: “*Do not know*”. Both questions have been drawn from The Danish Value Survey, which is a part of the international survey The European Values Survey (EVS) (Værdiundersøgelse 2008). The variables from the Danish Value Survey have been adapted to match a Danish context (e.g. the church/mosque attendance variable excluded weddings, funerals and christenings, as most Danes would attend these occasions for cultural reasons, even if they are not religious). The variables in the present study were dichotomized based on whether the respondents were generally open or closed to transcendence, which was defined based on the degree of prayer/meditation practice and church/mosque attendance. Option 1, 2 and 3 were considered open to prayer/meditation practice or church/mosque attendance, whereas option 4 was considered the opposite. Option 5 was excluded from the statistical analysis and presentation of data. The basis for excluding option 5 (“*Do not know*”) is due to lack of knowledge, whether this group of respondents were generally open or closed to transcendence.

### Health-Related Risk Behaviour

From the questionnaire, the following self-reported information is available: dietary habits, smoking status, alcohol consumption, physical activity and BMI.

#### Dietary Habits

Dietary habits were measured with four different items: intake of vegetables, fruit, fish and sweets (Socialstyrelsen 2011). Based on the four questions, a total diet score was calculated. The highest score of each question constituted the healthiest option. The highest possible total diet score was 12. A score of four or less was considered an unhealthy dietary pattern, whereas a score of five or above was considered a healthy dietary pattern (Socialstyrelsen 2011).

#### Smoking Status

Smoking status was obtained through one question (Martinez et al. 2008), and categorized as daily smoker versus all other categories of smoking (including *“occasional smoking”, “stopped *< *6* *months ago”, “stopped *> *6* *months ago”* and *“never smoked”*).

#### Alcohol Consumption

Alcohol consumption was measured by intake of alcoholic beverages during a typical week. In this study, a high-risk limit of alcohol consumption for men are 21 units or above per week and for women 14 units or above per week, based on recommendations from The Danish Health Authority (Sundhedsstyrelsen 2017).

#### Physical Activity

Information on physical activity was based on one question, considering the respondents’ level of physical activity during the last year (Christensen et al. 2004). Respondents, who reported that they primarily have sedentary activities like reading, watching television or other such activities, were categorized as physical inactive, whereas the rest were categorized as physical active (including *“Participate in sports competition and do sports several times a week”, “Do recreational sports or perform heavy gardening or similar at least 4* *h a week”* and *“Walking, cycling or other light exercise at least 4* *h a week”)* (Sundhedsstyrelsen 2016).

#### Body Mass Index (BMI)

The respondents’ BMI was calculated based on self-reported height and weight. In accordance with the recommendations from the World Health Organization on categorization of BMI, overweight was categorized as a BMI on 25 or above, whereas a BMI under 25 was categorized as not overweight (WHO 2018).

### Covariates

Previous research has shown that gender, age, country of origin, highest attained educational level, employment status and cohabitational status act as potential confounders for the association of interest (Glaeser and Sacerdote 2008; Hvidtjorn et al. 2014; Powell et al. 2003). Therefore, the above-mentioned covariates were retrieved from Statistics Denmark and adjusted for in the analysis. Age was treated as age-groups in the intervals: “*29*–*34* *years*”, “*35*–*39* *years*”, “*40*–*44* *years*”, “*45*–*49* *years*”, “*50*–*54* *years*” and “*55*–*60* *years*”. Country of origin was categorized as “*Denmark*” and “*Not Denmark*”. Highest attained educational level was categorized as “> *15* *years*”, “*10*–*15* *years*” and “≤ *10* *years*”. Employment status was categorized as “*Employee*”, “*Self*-*employed*” and “*Others*”, where the latter covers, among others, receivers of social security, post-employment benefits and state education grant. Cohabitational status was categorized as “*Cohabiting*” and “*Single*”.

## Statistical Analysis

The correlation between the two measures of religiosity and the different measures of health-related risk behaviours were analysed by logistic regression models, estimating odds ratios (ORs) and 95% confidence intervals (CIs). The risk factor outcome included the following health-related risk behaviours: unhealthy dietary pattern, daily smoking, alcohol consumption above the high-risk limit, physical inactivity and overweight (BMI ≥ 25). Bivariate (unadjusted) and multivariate (adjusted) analyses were performed for all correlations of interest. In the multivariate analysis, the following variables were included: gender, age group, country of origin, level of education, employment status and cohabitation status. The latter four were obtained from Statistics Denmark. Respondents who did not have full information available were excluded. All statistical analysis was conducted in Stata version 15.1.

## Results

**Fig. 1:**
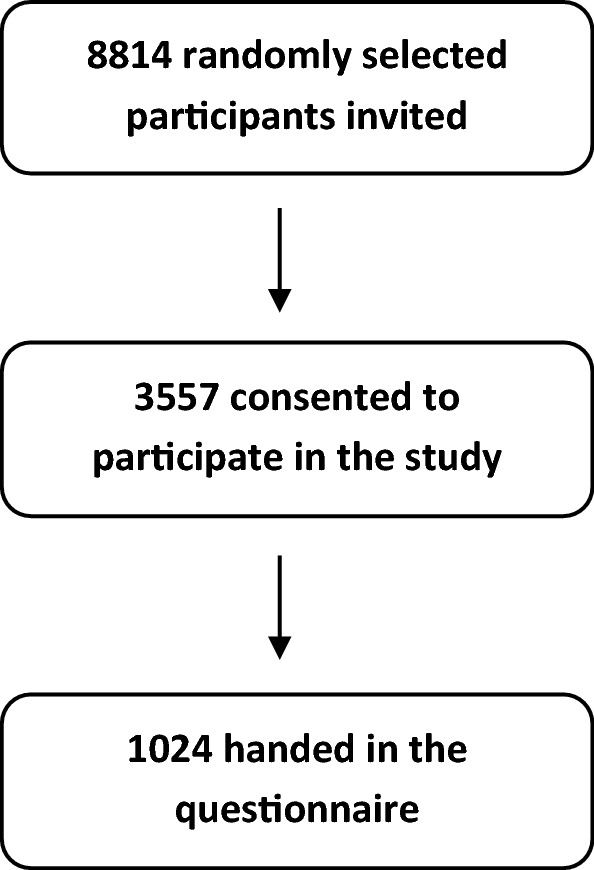
Flowchart over participants in the study

Among the 8814 patients invited, 3557 accepted to participate in the study and 1024 handed in the questionnaire on lifestyle and religiosity (Fig. 1). Full information regarding prayer/meditation practices were available from 958 respondents. Among them, 45.2% (*n *= 433) were open towards prayer/meditation practice. On church/mosque attendance full information was available from 986 respondents. Of these, 49.5% (*n *= 488) were open towards attending the mentioned religious settings. The respondents who were open towards either prayer/meditation practice or church/mosque attendance were more likely to be female, from the older age group, with high educational level and cohabiting (Tables 1, 2).

**Table 1:** Respondents characteristics regarding prayer/meditation practice

	Total	Closed towards prayer/meditation practice	Open towards prayer/meditation practice
Total (*n*)	958	525	433
Gender			
Women [*n*, (%)]	504 (52.6)	224 (42.7)	280 (64.7)
Men [*n*, (%)]	454 (47.4)	301 (57.3)	153 (35.3)
Age group			
55–60 years [*n*, (%)]	252 (26.3)	127 (24.2)	125 (28.9)
50–54 years [*n*, (%)]	310 (32.4)	169 (32.2)	141 (32.6)
45–49 years [*n*, (%)]	152 (15.9)	87 (16.6)	65 (15.0)
40–44 years [*n*, (%)]	114 (11.9)	65 (12.4)	49 (11.3)
35–39 years [*n*, (%)]	79 (8.2)	45 (8.6)	34 (7.8)
29–34 years [*n*, (%)]	51 (5.3)	32 (6.0)	19 (4.4)
Country of origin			
Denmark [*n*, (%)]	940 (98.1)	521 (99.2)	419 (96.8)
Not Denmark [*n*, (%)]	18 (1.9)	4 (0.8)	14 (3.2)
Level of education			
> 15 years [*n*, (%)]	298 (31.1)	116 (22.1)	182 (42)
10–15 years [*n*, (%)]	503 (52.5)	307 (58.5)	196 (45.3)
≤ 10 years [*n*, (%)]	157 (16.4)	102 (19.4)	55 (12.7)
Cohabitation status			
Cohabiting [*n*, (%)]	768 (80.2)	416 (79.2)	352 (81.3)
Single [*n*, (%)]	190 (19.8)	109 (20.8)	81 (18.7)
Employment status			
Employee [*n*, (%)]	807 (84.2)	456 (86.9)	351 (81.1)
Self-employed [*n*, (%)]	48 (5.0)	21 (4.0)	27 (6.2)
Other^a^ [*n*, (%)]	103 (10.8)	48 (9.1)	55 (12.7)
Dietary pattern			
Healthy [*n*, (%)]	760 (79.3)	394 (75.0)	366 (84.5)
Unhealthy [*n*, (%)]	198 (20.7)	131 (25.0)	67 (15.5)
Smoking status			
Not smoker [*n*, (%)]	874 (91.2)	470 (89.5)	404 (93.3)
Daily smoker [*n*, (%)]	84 (8.8)	55 (10.5)	29 (6.7)
Alcohol consumption			
Under the high-risk limit [*n*, (%)]	936 (97.7)	511 (97.3)	425 (98.2)
Above the high-risk limit [*n*, (%)]	22 (2.3)	14 (2.7)	8 (1.8)
Physical activity			
Regular physical activity [*n*, (%)]	832 (86.8)	450 (85.7)	382 (88.2)
Physical inactivity [*n*, (%)]	126 (13.2)	75 (14.3)	51 (11.8)
BMI			
< 25 [*n*, (%)]	414 (43.2)	205 (39.0)	209 (48.3)
≥ 25 [*n*, (%)]	544 (56.8)	320 (61.0)	224 (51.7)

^a^Other covers receivers of social security, post-employment benefits, state education grant and others

**Table 2:** Respondents characteristics regarding church/mosque attendance

	Total	Closed towards Church/mosque attendance	Open towards Church/mosque attendance
Total (*n*)	986	498	488
Gender			
Women [*n*, (%)]	518 (52.5)	224 (45.0)	294 (60.2)
Men [*n*, (%)]	468 (47.5)	274 (55.0)	194 (39.8)
Age group			
55–60 years [*n*, (%)]	258 (26.2)	136 (27.3)	122 (25.0)
50–54 years [*n*, (%)]	321 (32.5)	163 (32.7)	158 (32.4)
45–49 years [*n*, (%)]	159 (16.1)	70 (14.1)	89 (18.2)
40–44 years [*n*, (%)]	117 (11.9)	59 (11.9)	58 (11.9)
35–39 years [*n*, (%)]	81 (8.2)	38 (7.6)	43 (8.8)
29–34 years [*n*, (%)]	50 (5.1)	32 (6.4)	18 (3.7)
Country of origin			
Denmark [*n*, (%)]	969 (98.3)	490 (98.4)	479 (98.2)
Not Denmark [*n*, (%)]	17 (1.7)	8 (1.6)	9 (1.8)
Level of education			
> 15 years [*n*, (%)]	305 (30.9)	123 (24.7)	182 (37.3)
10–15 years [*n*, (%)]	519 (52.7)	272 (54.6)	247 (50.6)
≤ 10 years [*n*, (%)]	162 (16.4)	103 (20.7)	59 (12.1)
Cohabitation status			
Cohabiting [*n*, (%)]	797 (80.8)	386 (77.5)	411 (84.2)
Single [*n*, (%)]	189 (19.2)	112 (22.5)	77 (15.8)
Employment status			
Employee [*n*, (%)]	830 (84.2)	423 (84.9)	407 (83.4)
Self-employed [*n*, (%)]	50 (5.0)	17 (3.4)	33 (6.8)
Other^a^ [*n*, (%)]	106 (10.8)	58 (11.7)	48 (9.8)
Dietary pattern			
Healthy [*n*, (%)]	785 (79.6)	361 (72.5)	424 (86.9)
Unhealthy [*n*, (%)]	201 (20.4)	137 (27.5)	64 (13.1)
Smoking status			
Not smoker [*n*, (%)]	902 (91.5)	441 (88.6)	461 (94.5)
Daily smoker [*n*, (%)]	84 (8.5)	57 (11.4)	27 (5.5)
Alcohol consumption			
Under the high-risk limit [*n*, (%)]	963 (97.7)	483 (97.0)	480 (98.4)
Above the high-risk limit [*n*, (%)]	23 (2.3)	15 (3.0)	8 (1.6)
Physical activity			
Regular physical activity [*n*, (%)]	854 (86.6)	417 (83.7)	437 (89.5)
Physical inactivity [*n*, (%)]	132 (13.4)	81 (16.3)	51 (10.5)
BMI			
< 25 [*n*, (%)]	422 (42.8)	201 (40.4)	221 (45.3)
≥ 25 [*n*, (%)]	564 (57.2)	297 (59.6)	267 (54.7)

^a^Other covers receivers of social security, post-employment benefits, state education grant and others

As shown in Table 3, the respondents who reported to be generally open towards prayer/meditation practice have lower odds for an unhealthy dietary pattern (OR 0.6, CI 0.4–0.8) and overweight (BMI ≥ 25) (OR 0.7, CI 0.5–0.9) compared to individuals who do not engage in any kind of praying/meditation practice. The results were only statistically significant in the bivariate analysis. After stratifying by gender, lower odds for an unhealthy dietary pattern (OR 0.6, CI 0.3–0.9) were seen among women, in the bivariate analysis. Among the respondents, who reported to be generally open towards church/mosque attendance, lower odds for an unhealthy dietary pattern (OR 0.4, CI 0.3–0.6), daily smoking (OR 0.6, CI 0.3–0.9) and physical inactivity (OR 0.6, CI 0.4–0.9) were observed compared to individuals who do not attend church/mosque (Table 4), the latter only in the bivariate analysis. After stratifying by gender, both sexes had lower odds for an unhealthy dietary pattern; however, the odds were slightly lower for women than for men. Beyond that, only women had lower odds for daily smoking (OR 0.4, CI 0.2–0.9) (unadjusted model).

**Table 3:** Logistic regression analysis over the correlation between prayer/meditation practice and unhealthy risk behaviours, for the overall study population and stratified by gender

Prayer/meditation practice	Overall		Women		Men	
	Unadjusted analyses	Adjusted analyses^a^	Unadjusted analyses	Adjusted analyses^b^	Unadjusted analyses	Adjusted analyses^b^
	OR (95% CI)	OR (95% CI)	OR (95% CI)	OR (95% CI)	OR (95% CI)	OR (95% CI)
Unhealthy dietary pattern	**0.6 (0.4; 0.8)**	0.7 (0.5; 1.0)	**0.6 (0.3; 0.9)**	0.7 (0.4; 1.2)	0.7 (0.5; 1.1)	0.8 (0.5; 1.3)
Daily smoking	0.6 (0.4; 1.0)	0.7 (0.4; 1.2)	0.6 (0.3; 1.3)	0.6 (0.3; 1.3)	0.8 (0.4; 1.4)	0.8 (0.4; 1.6)
Alcohol consumption above the high-risk limit	0.7 (0.3; 1.7)	1.0 (0.4; 2.5)	1.1 (0.2; 4.8)	1.2 (0.2; 5.5)	0.7 (0.2; 2.3)	0.9 (0.3; 3.2)
Physical inactivity	0.8 (0.5; 1.2)	0.9 (0.6; 1.4)	0.8 (0.5; 1.4)	0.9 (0.5; 1.7)	0.9 (0.5; 1.6)	1.1 (0.6; 1.9)
Overweight (BMI ≥ 25)	**0.7 (0.5; 0.9)**	0.8 (0.6; 1.1)	0.8 (0.6; 1.2)	0.9 (0.6; 1.4)	0.7 (0.4; 1.0)	0.7 (0.5; 1.1)

Number in bold indicated statistical significance (*p* < .05)

^a^Adjusted for gender, age group, country of origin, level of education, employment status and cohabitation status

^b^Adjusted for age group, country of origin, level of education, employment status and cohabitation status

**Table 4:** Logistic regression analysis over the correlation between church/mosque attendance and unhealthy risk behaviours, for the overall study population and stratified by gender

Church/mosque attendance	Overall		Women		Men	
	Unadjusted analyses	Adjusted analyses^a^	Unadjusted analyses	Adjusted analyses^b^	Unadjusted analyses	Adjusted analyses^b^
	OR (95% CI)	OR (95% CI)	OR (95% CI)	OR (95% CI)	OR (95% CI)	OR (95% CI)
Unhealthy dietary pattern	**0.4 (0.3; 0.6)**	**0.4 (0.3; 0.6)**	**0.4 (0.2; 0.6)**	**0.3 (0.2; 0.6)**	**0.5 (0.3; 0.7)**	**0.5 (0.3; 0.8)**
Daily smoking	**0.5 (0.3; 0.7)**	**0.6 (0.3; 0.9)**	**0.4 (0.2; 0.9)**	0.5 (0.2; 1.0)	0.6 (0.3; 1.0)	0.7 (0.4; 1.3)
Alcohol consumption above the high-risk limit	0.5 (0.2; 1.3)	0.7 (0.3; 1.8)	1.0 (0.2; 4.6)	1.2 (0.3; 5.7)	0.5 (0.1; 1.4)	0.6 (0.2; 1.9)
Physical inactivity	**0.6 (0.4; 0.9)**	0.7 (0.5; 1.1)	0.7 (0.4; 1.1)	0.7 (0.4; 1.3)	0.6 (0.4; 1.0)	0.7 (0.4; 1.2)
Overweight (BMI ≥ 25)	0.8 (0.6; 1.1)	1.0 (0.7; 1.3)	1.0 (0.7; 1.4)	1.1 (0.8; 1.6)	0.8 (0.5; 1.1)	0.8 (0.5; 1.2)

Number in bold indicated statistical significance (*p* < .05)

^a^Adjusted for gender, age group, country of origin, level of education, employment status and cohabitation status

^b^Adjusted for age group, country of origin, level of education, employment status and cohabitation status

## Discussion

Results from the multivariate analysis demonstrate that church/mosque attenders had significantly healthier dietary patterns and were less likely to be daily smokers. Furthermore, it seems that church/mosque attenders may be more physically active. Based on results from the bivariate analysis, individuals participating in prayer/meditation practice may have a healthier dietary pattern and be less often overweight. The results thereby support the findings from several other studies (Brown et al. 2014; Kim and Sobal 2004; Kobayashi et al. 2015; Koenig et al. 2012), concluding that individuals participating in religious activities are more likely to have healthy lifestyle habits. Therefore, the present results indicate that citizens living in more secular cultures may have similar patterns in terms of healthy lifestyle as those found in more religious cultures, such as the USA and different religious minority cultures.

Several studies report positive associations between religiosity and diet quality (Koenig et al. 2012). A possible explanation for this association lies within the different religious orientations and their lifestyle recommendations and ordinances. An interesting point is that most of the studies showing an association between religiosity and healthy diet are conducted among, e.g. Jews or religious minorities, such as Adventists (Phillips 1975; Shmueli and Tamir 2007; Lau Caspar Thygesen et al. 2012b). Our study population differs significantly from religious minorities, since the respondents form part of a mainstream, culture which we assume take no part in a religious doctrine with specific eating rules, such as Ramadan or kosher food.

In the present study, church/mosque attendance was associated with lower odds of being a daily smoker. Overall, our results support the findings from other studies in this regard (Bailey et al. 2015; Brown et al. 2014). The results of this study support the hypothesis that religious community life and ritual attendance can help strengthen the individual not to engage in risk behaviours such as smoking (Hall et al. 2008). A reason for this could be that different norms and values embedded in the various religious communities induce the individual to have a non-smoking behaviour. As shown in Table 1 and 2 the overall sample size is limited, which may have influenced the results. The representativeness of the sample may also have influenced the results. The latter is discussed more in the section “Strength and limitations”.

In the present study, bivariate analyses revealed lower odds of physical inactivity among the group of respondents attending church/mosque compared to those not attending. No such association was observed between prayer/meditation practice and physical activity. A previous study found that the degree of praying is associated with moderately increased levels of physical activity among men and that religious commitment, especially money donated to religion, was associated with moderate to vigorous physical activity among women (Kim and Sobal 2004). A reason why we did not find any significant association between religiosity and physical activity in the multivariate analyses and after gender stratification may be due to the limited sample size.

Based on results from the bivariate analysis, respondents who engage in prayer/meditation practice seem to have lower odds of being overweight. Previous research on the association between religiosity and body weight has shown inconsistent results (Yeary et al. 2017). A study conducted among Indian immigrants in the USA found that religious individuals have significantly higher odds of being overweight, compared to individuals who were less religious (Bharmal et al. 2013). Opposite results were found by Reeves et al. (2012) studying the relationship between faith and overweight among African Americans. Bharmal et al. (2013) explained the association between religiosity and higher bodyweight by the lower prevalence of smokers, since it is well known that nicotine act as an appetite suppressant. One possible explanation for these inconsistent results might be differences in ethnicity and could thereby be explained by demographics.

In contrast to the existing literature suggesting that religious individuals have a lower alcohol intake compared to non-religious individuals (Koenig et al. 2012), no such association occurs in our study. The reason for the missing association may, however, be due to the small number of respondents reporting high-risk alcohol intake. This could also be due to a possible reporting bias, which naturally can occur when using self-administered questionnaires.

### Strengths and Limitations

The present study comprises a relatively sparse number of participants compared to other similar studies (Obisesan et al. 2006). In addition, the possibility for selection and information bias must be carefully considered in cross-sectional studies using self-administered questionnaires. The representativeness of the sample may have been influenced as citizens consenting to participate in the TOF study was more likely to be women, older citizens, citizens with higher socio-economic status (SES), and citizens not diagnosed with or in treatment of certain lifestyle-related diseases (Larsen et al. 2018a, b). This could indicate some selection bias, and a possibility for differential misclassification, which may affect the external validity of the study, leading to an underestimation of the true association. As the study uses self-administered questionnaire, the possibility for recall-bias and reporting bias naturally occurs (Fayers and Machin 2009). Some associations reached statistical significance in the unadjusted models, only. This may be explained by confounding or the relatively large numbers of variables included which may result in a lack of statistical power (Peduzzi et al. 1996). Despite these limitations, the present study population is the most representative Danish population studied regarding religiosity and lifestyle.

The choice of variables describing measures of religiosity in the present study poses strengths as well as limitations. Items on church/mosque attendance as well as prayer/meditation practice have been widely used in previous international studies, such as the Danish Value Survey (Værdiundersøgelse 2008). In addition, in the field of religiosity very few questionnaires have been validated, due to the complexity (Hall et al. 2008).

In terms of choice of dichotomization of response categories, our aim and hence choice of analysis strategy allowed to compare those who are open and those who are closed to transcendence [to use Charles Taylor’s terms (Pesut 2010)]. We therefore chose to compare respondents reporting some or much religiosity to none, rather than much versus little religiosity (measured on attendance).

A cross-sectional study, like the present study, only shows a snapshot of the study population. Therefore, it is not possible to evaluate whether religiosity influences the health habits, or whether individuals with existing healthy habits engage more in religious activities. A longitudinal population-based cohort study might be ideal and could help clarifying the above-mentioned.

A strength of the present study is the study population which consists of young to middle-aged (29–60-years-old) Danish citizens, a segment that has never been studied before regarding religiosity and health-related risk behaviours. Moreover, this study includes more detailed information on religiosity and several aspects of lifestyle habits such as dietary pattern, smoking, alcohol intake, physical activity and overweight, which we expect will give a good overall status of the individual’s actual lifestyle.

## Conclusion and Future Perspectives

This study showed that church/mosque attendance and prayer/meditation practice were statistically significantly correlated with some of the variables pertaining to a healthy lifestyle. The results demonstrate a possible connection between religiosity and healthy lifestyle, to which healthcare professionals should pay more attention, particularly in relation to, e.g. prevention of lifestyle-related diseases. Clinicians could embrace this by identifying the individuals’ religiosity and practice as a part of the clinical prevention dialogue between, e.g. the general practitioner and patient. Furthermore, knowledge of patients’ religiosity and practice might help clinicians to understand some of the lifestyle choices the patients’ make and support the religious resources that aid them in coping with their disease crisis. In general, our study calls for further research identifying the social and cultural mechanisms behind these findings. More work should be done on the possible role of different underlying factors, such as how the experiences of social connectedness and community feeling among religious individuals might influence health-related risk behaviours. Furthermore, it would be interesting to conclude a timewise sequence of events when investigating further the theoretical dichotomy of restful and crisis religiosity, where restful religiosity is associated with good health opposed to crisis religiosity (Ahrenfeldt et al. 2017; Hvidt et al. 2017). Restful religiosity is characterized as a longstanding faith that is deeply rooted in the individual, whereas the opposite, crisis religiosity, is characterized as a type of faith triggered by stress and where the individual finds an increased belief in religion during a crisis. It would be interesting to investigate how restful and crisis religiosity influence health-related risk behaviours.
